# CAD-Skin: A Hybrid Convolutional Neural Network–Autoencoder Framework for Precise Detection and Classification of Skin Lesions and Cancer

**DOI:** 10.3390/bioengineering12040326

**Published:** 2025-03-21

**Authors:** Abdullah Khan, Muhammad Zaheer Sajid, Nauman Ali Khan, Ayman Youssef, Qaisar Abbas

**Affiliations:** 1 Department of Computer Software Engineering, Military College of Signals, National University of Science and Technology, Islamabad 44000, Pakistan; akhan.mscs2021mcs@student.nust.edu.pk (A.K.); msajid.msse-27mcs@student.nust.edu.pk (M.Z.S.); nauman@mcs.edu.pk (N.A.K.); 2 Department of Computers and Systems, Electronics Research Institute, Cairo 12622, Egypt; 3 College of Computer and Information Sciences, Imam Mohammad Ibn Saud Islamic University (IMSIU), Riyadh 11432, Saudi Arabia; qaabbas@imamu.edu.sa

**Keywords:** skin lesion classification, artificial intelligence in healthcare, deep learning, convolutional neural network (CNN), quantum support vector machine (QSVM), Internet of Things (IoT)

## Abstract

Skin cancer is a class of disorder defined by the growth of abnormal cells on the body. Accurately identifying and diagnosing skin lesions is quite difficult because skin malignancies share many common characteristics and a wide range of morphologies. To face this challenge, deep learning algorithms have been proposed. Deep learning algorithms have shown diagnostic efficacy comparable to dermatologists in the discipline of images-based skin lesion diagnosis in recent research articles. This work proposes a novel deep learning algorithm to detect skin cancer. The proposed CAD-Skin system detects and classifies skin lesions using deep convolutional neural networks and autoencoders to improve the classification efficiency of skin cancer. The CAD-Skin system was designed and developed by the use of the modern preprocessing approach, which is a combination of multi-scale retinex, gamma correction, unsharp masking, and contrast-limited adaptive histogram equalization. In this work, we have implemented a data augmentation strategy to deal with unbalanced datasets. This step improves the model’s resilience to different pigmented skin conditions and avoids overfitting. Additionally, a Quantum Support Vector Machine (QSVM) algorithm is integrated for final-stage classification. Our proposed CAD-Skin enhances category recognition for different skin disease severities, including actinic keratosis, malignant melanoma, and other skin cancers. The proposed system was tested using the PAD-UFES-20-Modified, ISIC-2018, and ISIC-2019 datasets. The system reached accuracy rates of 98%, 99%, and 99%, consecutively, which is higher than state-of-the-art work in the literature. The minimum accuracy achieved for certain skin disorder diseases reached 97.43%. Our research study demonstrates that the proposed CAD-Skin provides precise diagnosis and timely detection of skin abnormalities, diversifying options for doctors and enhancing patient satisfaction during medical practice.

## 1. Introduction

The practice of the classification of medical images is a critical activity and necessary research area. This research area is particularly important in the diagnosis of cancer, which can affect the skin [1], brain [2,3], lungs [4], breast [5], and eyes [6,7]. Skin cancer is considered one of the most common diseases present in the world these days, as the body’s largest organ is the skin [8]. Based on data from the World Health Organization, the United States reported an average of 104,350 cases of skin cancer in 2019; 11,650 of these cases resulted in death [9]. A total of 196,060 new cases of skin cancer were predicted for 2020, with an estimated 40,160 cases in men and 60,190 cases in women [10]. These figures suggest a significant escalation in cases, though the death rate is expected to decline by over 5.3% [11]. The main two categories of skin cancer are non-melanoma and melanoma cancer categories [12].

Melanoma, which has been proven to be the most severe kind of skin cancer, has been undergoing a sharp increase in number of cases both in developed and undeveloped countries [13]. It was estimated that there would be 106,110 new cases of melanoma in the United States in 2021, and the disease was expected to cause 7180 fatalities [14]. These numbers illustrate the necessity of early and accurate identification to facilitate patients’ having a milder course of the disease. Melanoma originates in melanocytes, the cells that can proliferate excessively to form a malignant tumor [15]. It predominantly affects the sun-exposed skin regions, including the hands, face, neck, and lips [16]. Early detection significantly improves the prospects for successful treatment; otherwise, the cancer may spread, leading to severe consequences and potentially death. However, even with the help of specialized computer-aided detection (CAD) tools that were introduced in the literature, the diagnosis of skin cancer in its early stages can still be difficult for specialists [17]. In the case of melanoma, there are certain characteristics such as its complex patterns and uneven pigment distribution that can help diagnose the disease [18]. Also, other methods exist for accurate diagnosis of melanoma, such as non-invasive dermoscopy. This method aids dermatologists in visualizing suspicious areas beneath the skin for accurate diagnosis of the disease. Dermatologists have become much more skilled at differentiating between melanoma and other forms of skin cancer thanks to the ABCD rule, the Menzies method, the seven-point checklist [19,20], and the CASH guideline [21]. In addition to being time-consuming, the diagnosis procedure is complicated and expensive because it relies on visual inspection [22]. In summary, the desire to develop automated systems that accurately detect and classify skin cancer has significantly grown. These processes can be illustrated by identifying lesions, recording their characteristics, and categorizing them. For this task, deep learning (DL) methods show a high degree of proficiency in medical imaging, including the categorization of skin cancer [23,24,25].

**Figure 1:**
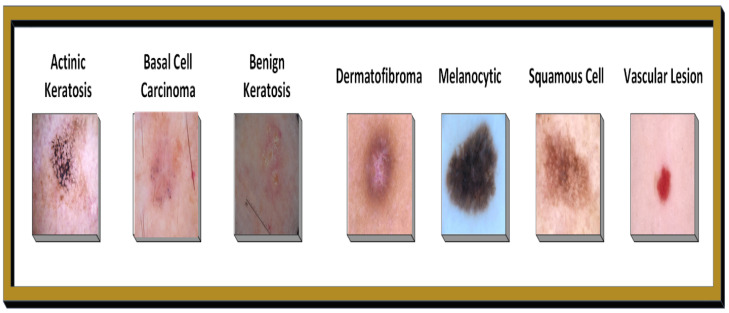
Skin disease images by severity level.

Figure 1 illustrates the Skin Image Categorization based on severity levels.

Table 1 shows the records of clinical findings in relation to skin disorders.

This paper proposes a novel deep learning framework for the classification of skin disease that provides intelligent and economical skin disease monitoring and diagnosis. A survey of several DL algorithms used in skin cancer detection and classification is presented in this research that demonstrates the expanding range of methods as the field of research evolves. The surveyed systems involve a variety of architectures and models, namely, convolutional neural networks (CNNs), residual deep networks, transfer learning, and an integrated approach that comprises deep feature fusion. Particularly, a number of models are discussed, including AlexNet, VGG16, InceptionNet, DenseNet, PNASNet-5, ResNet-101, MobileNetV2, and LeNet-5, to name just a few. In addition, hybrid schemes are presented that combine CNNs with other types of neural networks or machine learning techniques like SVM.

**Figure 2:**
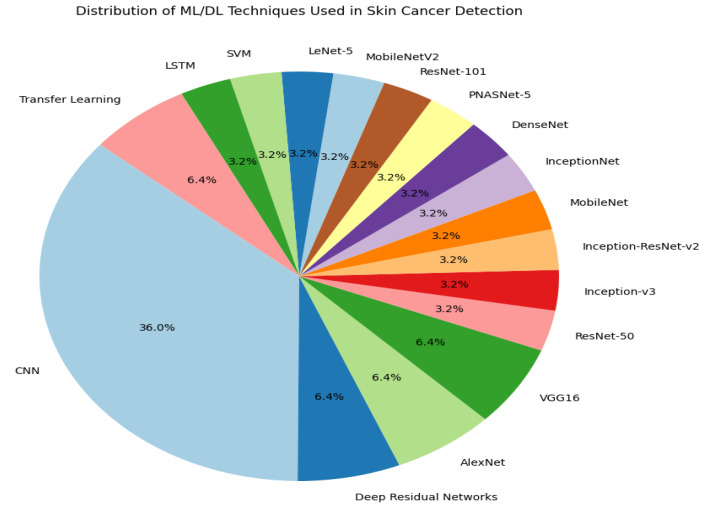
Pie chart illustrating the distribution of deep learning and machine learning methods for identifying and classifying skin cancer.

In summary, the literature survey in this paper mentions at least 17 specific deep learning models used in skin cancer classification and detection. These methods are critical for advancing skin cancer’s diagnostic accuracy and addressing the limitations of traditional clinical methods. Figure 2 shows a diverse range of models and approaches, with convolutional neural networks (CNNs) being the most frequently mentioned, reflecting their prominence in this field. This visual representation helps us to understand the variety and frequency of techniques used in recent studies.

### 1.1. Research Motivation

Skin cancer is widely recognized for its aggressive nature in terms of how quickly it spreads in the body and its high mortality rates on a global scale. Despite significant efforts and advancements made in various diagnostic strategies, the precise identification and classification of skin lesions continue to be challenging for healthcare professionals. For example, the complexity of melanoma lesions further adds to the challenge of diagnosing this type of cancer accurately in a clinical setting. The goal of this study is to develop a practical system for skin cancer diagnosis and classification in intelligent and cost-effective methods. The ultimate aim is to establish a dependable computerized system specifically tailored for detecting skin cancer that surpasses the capabilities of current computer-aided detection methods. By leveraging deep learning models, the goal is to improve the accuracy of skin cancer diagnosis.

### 1.2. Problem Statement

In the past several years, there has been a significant increase in the use of machine learning (especially deep learning) in the field of medical imaging, especially in the diagnosis and classification of diseases.

One research area of medical imaging is skin cancer classification. This research area needs novel ideas and methodologies in image processing and deep learning fields. The main challenge that faces research in this area is that the accuracy of current systems for segmenting and classifying skin cancer lesions is low. This is because of the broad range of forms and sizes presented by lesions, the low contrast of lesion areas, and the existence of irregular patterns of lesions. All these factors pose challenges to precise feature extraction from lesion areas. Another challenge that may face such systems is imbalanced datasets, where certain skin lesion classes have higher numbers than others. This can bias classification accuracy toward those classes. Another challenge that may face such classification models is irrelevant features that can also lead to classification errors. This problem can be solved by feature selection algorithms. Another challenge about skin cancer classification is that there are six different classes that need to be classified by the DL model. This work introduces a novel model to address these issues. The proposed model uses autoencoders for extracting features from images. Also, the work proposes a novel image processing algorithm for increasing the images’ quality in order to increase the accuracy of the proposed DL model.

### 1.3. Research Contribution

The proposed CAD-Skin framework integrates key features of CNN and autoencoder approaches, combining residual and densely connected encoder–decoder structures to enhance processing speed and alleviate overfitting, leading to improved classification accuracy. The contributions of this work can be summarized in the following points:

- A novel image processing algorithm is proposed in this work. The proposed algorithm consists of a series of preprocessing filters that are applied to all input images. These filters include spatiotemporal retinex, gamma correction, histogram equalization, unsharp masking, and contrast-limited adaptive histogram equalization. These techniques improve image detail, making it easier to accurately identify and classify skin diseases.
- Data augmentation techniques are employed to overcome the scarcity of the annotated dataset, generating numerous examples from limited labeled data. This approach ensures a comprehensive representation of various skin disease severities.
- Another contribution of this work is that the integration of a Quantum Support Vector Machine (QSVM) classifier enhances the final classification step, providing realistic confidence levels and robust handling of multi-class issues. This supports more accurate and reliable predictions of skin disease severity levels.

### 1.4. Paper Organization

The paper is organized as follows: Section 2 discusses the literature survey of relevant research articles. Section 3 discusses and explains the proposed methodology. Section 4 reports the results of the proposed work on a number of different experiments. Section 5 proposes the state-of-the-art comparison of the proposed model with different models from literature. Section 6 discusses the contribution of the work and significance of the results. Section 7 gives the most important conclusions of the work.

## 2. Literature Survey

The current skin cancer diagnostics techniques have proven insufficient, which means a more advanced automated system for the precise classification of skin cancer [23] is needed.

One of the most important traditional techniques for skin cancer diagnosis is dermoscopy. Dermoscopy (a method revolutionizing skin color lesion examination and research) enables an unprecedented look into skin lesions and helps with the precise separation of melanoma from the non-melanoma types. Using dermoscopy images and computers can contribute by providing diagnosis and classification of skin lesions [24,25,26]. Important steps of this procedure include lesion segmentation, feature extraction, lesion classification, and image processing.

However, the accurate classification of skin lesions poses some intractable challenges as discussed in the Introduction, hence the need for deep learning models. Very recently, the computer vision domain has revealed the significance of deep learning algorithms for image segmentation, detection, and classification, resulting in a high level of accuracy for complex problems [27].

Utilizing deep learning models has the capacity to become a new and powerful way to increase the precision and effectiveness of skin cancer diagnosis. Also, deep learning models can be powerful in the image segmentation task [28] needed for skin classification. There are a number of review articles that summarize the contribution of machine learning algorithms [13,29,30] in the skin classification domain. CNNs have been used since 2015 in Dermoscopic Image Analysis, and they have gained the most popularity as one of the most powerful methods for skin disease classification.

Codella et al. [31] studied the recognition of malignant skin lesions and evaluated and contrasted the performance of popular deep neural networks, including CNN models and deep residual networks.

Simon et al. [32] proposed a deep learning framework for segmenting and classifying skin lesions using 12 dermatologist-defined tissue classes, and then the features were used for final classifying by means of a deep CNN. This strategy demonstrated better efficiency than clinical methods, and a computerized system was able to attain 97.9% accuracy, contrasting with the clinical method’s 93.6%. Hamida et al. [33] proposed a new hybrid model that incorporates the strengths of random forest (RF) and deep neural network (DNN) algorithms. The system employs data augmentation and balancing techniques to enhance model performance.

Amin et al. [34] suggested a combined strategy for deep feature fusion that includes preprocessing, segmentation, and feature extraction. This work was exploiting the Otsu algorithm and biorthogonal 2D wavelet transform for lesion segmentation with further feature extraction performed through pretrained AlexNet and VGG16 models.

Al.masni et al. [35] suggested a deep learning framework that combines segmentation and classification of skin lesions, using a resolution convolutional network (FRCN) for segmentation followed by classification through various deep learning models. Their model showed promising accuracies across various datasets, with ResNet-50 demonstrating exceptional performance.

Pacheco et al. [36] evaluated thirteen different deep learning networks, finding the SENet CNN and Adam optimization to be the most effective, achieving 91% performance in the ISIC2019 dataset. Farooq et al. [37] improved classification performance to 86% using MobileNet and InceptionNet on the updated Kaggle dataset for skin cancer.

Lui et al. [38] employed a traditional deep learning approach with DenseNet and ResNet, along with the MFL module, attaining an 87% accuracy on the ISIC 2017 dataset. Using a linear SVM and Feedforward Neural Network (FNN), Pedro et al. [39] created a classification model that achieved 90% accuracy on the dermo fit dataset. Milton et al. [40] comprehensively evaluated various deep learning models on the ISIC-2018 dataset, with PNASNet-5 achieving the highest performance of 76%.

Khatib et al. [41] introduced a ResNet-101 Architecture for skin lesion classification, achieving 90% accuracy on the PH2 database through fine-tuned CNN models and transfer learning.

Almaraz et al. [42] presented a study which applied the ABCD rule with hand-crafted features along with MobileNetV2 to obtain 92.4% accuracy using the random forest classifier fitted on the HAM10000 dataset.

The deep learning-based algorithm of Shanthi et al. [43] managed to reach high detection accuracy rates in four specific skin conditions, acne, keratosis, Eczema herpeticum, and urticaria, based on DermNet’s data that reached 98.6% and 99.04% during the testing process. In addition, Jessica S. Velasco et al. [44] and Agarwal et al. [45] used transfer learning to increase the prediction accuracy in different datasets; the models have considerably amplified the categorization power of the model as they employ relevant dermatological datasets and pretrained CNNs that convey the granular features that usually relate to skin conditions.

Bhadoria et al. [46] illustrated that transfer learning could be used effectively by employing pretrained CNNs in such a fashion to give a boost to their performance in the classification of skin diseases. They inferred that the design of an effective image processing algorithm could help achieve good results in the classification of skin diseases. These methods become useful in dermatological applications, where datasets are lacking and vary in size.

Further, Alenezi et al. [47] and Hameed et al. [48] implemented pretrained CNNs and SVMs for disease detection, showcasing the model’s efficiency across different datasets and enhancing feature extraction through attention mechanisms. Min Chen et al. [49] proposed a real-time, dynamic system for detecting skin diseases, integrating self-learning and extensive data collection for improved user interaction, utilizing a data filter algorithm with information entropy to refine data quality. They employed LeNet-5, AlexNet, and VGG-16 CNN models for classification, evaluating the system’s reliability through computational and transmission delays, finding delays of 63 ms and 75 ms for LeNet and AlexNet, respectively.

Liao et al. [50] introduced a deep CNN-based method using advanced architectures like VGG-16, VGG-19, and GoogleNet, with experiments conducted on Dermnet and OLE datasets to compare model performance. Srinivasu et al. [51] combined MobileNet V2 and LSTM for skin disease classification on the HAM10000 dataset, achieving an accuracy of 85% and proposing a web application for skin reduction classification. K. S. Rao et al. [52] focused on developing an automated system for early skin lesion assessment, employing CNNs to detect common skin lesions with a 93% accuracy rate across seven classes on over 10,000 images.

In this work [53], an advanced semi-automated system for skin disease classification is proposed. The automated system consists of a feature enhancement algorithm (gradient contrast), a feature selection mechanism (revised anti-Lion algorithm), and an improved fine-tuning process of the deep learning network. The final system was able to attain a remarkable accuracy rate of 96.1% and 99.9% in both the cases of the ISIC2018 and ISIC2019 datasets. These outcomes are significant due to their marked difference over others and also bring forward the application of the proposed method in the improvement of the computing time as well as keeping low misdiagnosis. This study [54] proposes a highly sophisticated neural network model for the early detection and classification of different skin cancers, including melanoma, an aspect that should be addressed considering their high mortality rates and increased prevalence among the population. The first stage of this process includes the use of innovative image preprocessing combined with precision feature extraction and fusion. Second, hyperparameter optimization was performed with the genetic algorithm to increase the accuracy of the classifier. Finally, marine predator optimization identified and excluded the least important features, leading to the remarkable classification accuracies of 85.4% and 98.80% on the ISIC2018 and ISIC2019 datasets for each. These outcomes, when confirmed by comparative analysis, illustrate the capacity of the proposed framework to rise above performance and achieve a greater precision in the diagnosis of skin cancer and improve patient outcomes.

AlSadhan et al. [55] suggested a deep learning-powered image-based method to automatically identify and distinguish benign skin lesions from malignant ones; in particular, benign nevi, seborrheic keratosis, and malignant melanoma are the three most common lesions. Additionally, the authors investigated contemporary deep learning-based methods that focus on localization, classification, and recognition in order to identify skin lesions. In addition, YOLOv5 and YOLOv7 were assessed and contrasted for automatically classifying skin cancer lesions in the ISIC 2017 dataset. The findings determined in the paper reported that the most successful model was YOLOv7, which was further enhanced by using an appropriately augmented training set. A summary of these techniques is presented in Table 2, showcasing the diverse approaches and their outcomes. The literature survey shows that most of the previous work does not achieve high accuracies in the classification of skin lesions. Also, in the previous work, no one proposed using autoencoders as a feature extraction technique to increase the accuracy of the DL models. The proposed model in this work addresses this research gap by proposing a new model that uses autoencoders as a feature extraction technique and QSVM as a classification step.

## 3. Proposed Approach

This study concentrates on the investigation of applying autoencoders for feature extraction from medical images to increase the accuracy of DL models in disease classification. In this work, we concentrate on skin disease classification to show the high efficiency of autoencoders in extracting features from medical images. The proposed methodology in this work is meant to use the strengths of autoencoders in increasing the efficiency of the skin disease classification model.

**Figure 3:**
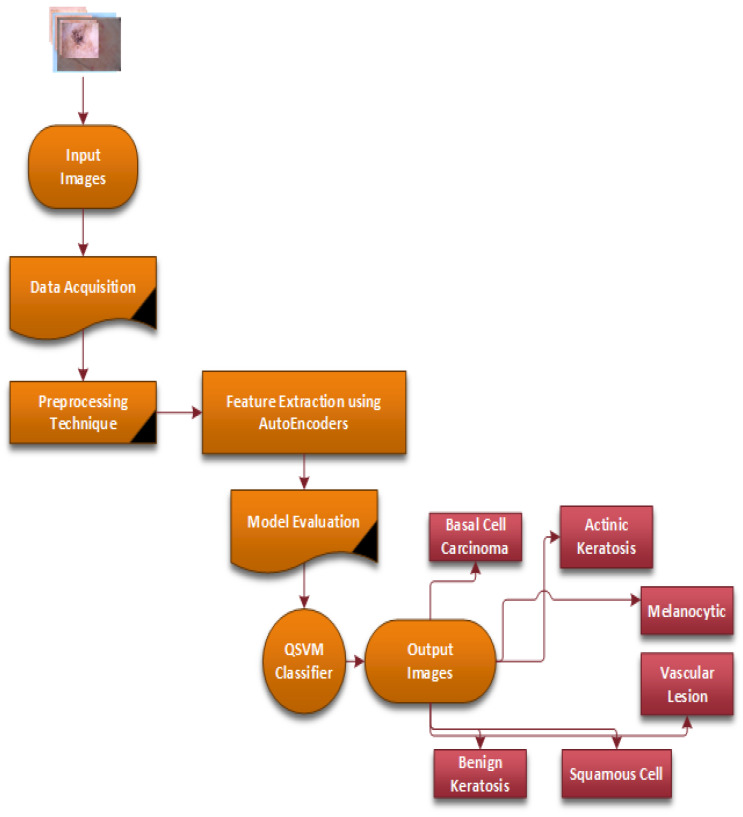
The complete steps of the proposed methodology of the CAD-Skin system.

Figure 3 provides a flow chart of the complete steps of the proposed CAD-Skin system. The next sections will discuss in detail each component of the methodology and its role in achieving the model goals. The next sections cover essential areas such as data acquisition, preprocessing, the model architecture’s main diagram, and the training of the model.

### 3.1. Data Acquisition and Preprocessing

**Figure 4:**
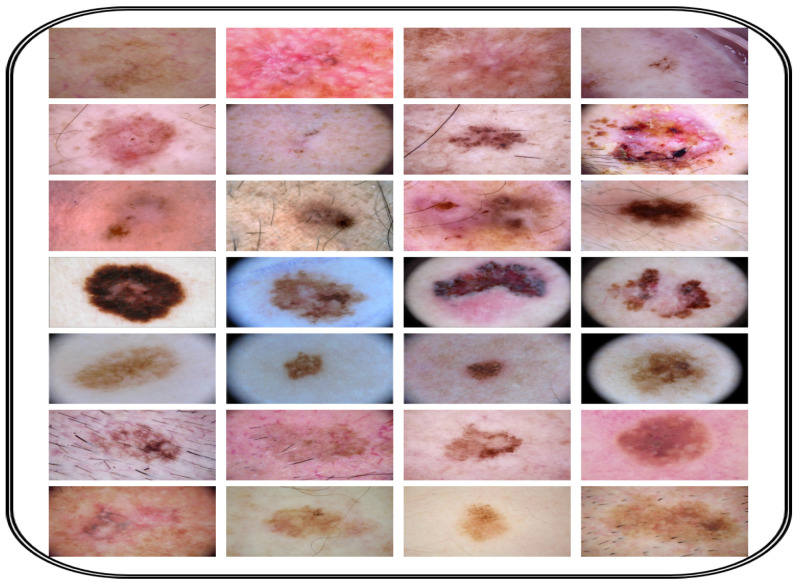
Image dataset collection for CAD-Skin model.

To develop and assess the CAD-Skin model, a comprehensive dataset named SkinDataSet [54] comprising 27,357 images was utilized along with images from prominent online sources. These images were accordingly saved as JPEG files at 1125 × 1264 resolution and were acquired during normal screenings. The dataset was meticulously curated with the assistance of expert dermatologists who manually categorized the skin images into high-resolution skin disease and non-disease groups. Figure 4 shows a number of images from the proposed dataset. Also, publicly available datasets (ISIC 2018 [56] and ISIC 2019 [56]) were used to further evaluate the proposed model. Table 3 presents the distribution of these two datasets that were combined to assemble the new dataset used for training and testing. The preparation of the image database is the main component of the first phase of the methodological process because the model should be both accurate and generalized. The dataset is arranged in a hierarchical folder structure, such that each sub-folder represents a separate category. This framework lends itself well to a multi-class categorization problem. The first step in the preprocessing is resizing all images to 256 × 256 dimension. This downsizing not only solves computational problems but ensures constancy from the dataset. The next step is revising and generating labels for each image. These labels show whether or not a certain disease is present in the image. The authors worked on ensuring an equal number of images represented each class from the dataset. This is performed to make sure that the dataset remains balanced and objective.

The implementation of the proposed model utilizes some python libraries like TensorFlow and keras to ease the preprocessing step of the model. In the first step of the implementation, the images are loaded from a predefined path, and each image is automatically combined with its label to simplify the overall classification process. The images are then grouped in 64 batches (this size has been selected to balance memory usage and model output). In addition to that, the dataset is partitioned into an 80% training set and 20% validation set with a fixed seed used to ensure the reproducibility of the process of shuffling and splitting the data. Critical here is the stratified splitting to assess the model’s ability to generalize beyond the training data for evaluation.

**Figure 5:**
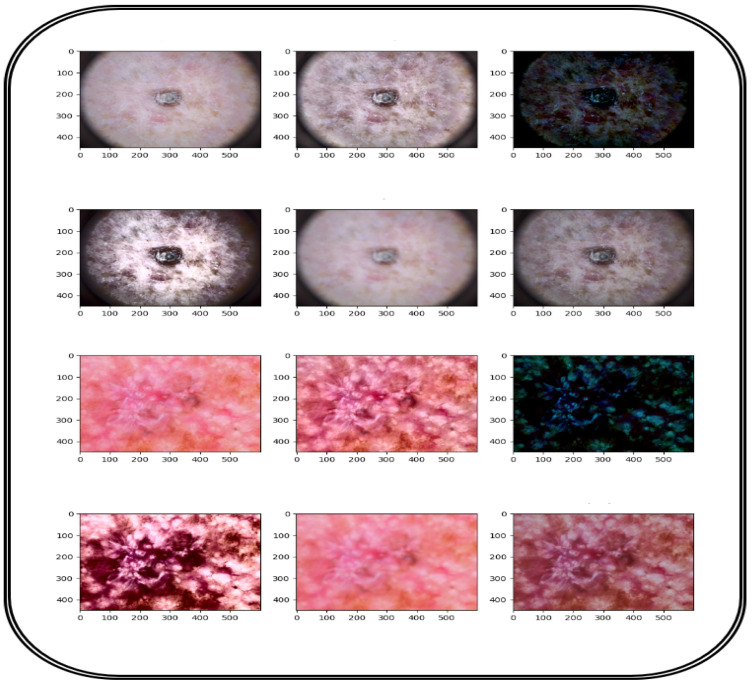
A visual result of image preprocessing to enhance contrast while adjusting noise pixels.

In the next step, preprocessing steps are applied. Table 4 shows these steps, which include first feature engineering data by the nominalization step and selecting the most critical features to increase the efficiency of the proposed model on the allocated dataset. Also, in the preprocessing stage, the images go through multiple image processing steps. These steps include removing irrelevant portions of the images, contrast enhancement for better brightness levels, quality improvement through multi-scale retinex, gamma correction, histogram equalization, unsharp masking, and contrast-limited adaptive histogram equalization methods. Figure 5 shows the effects of these preprocessing actions when applied to images prior to input into the proposed model. Cropping was essential to focus on significant parts of the image, while contrast adjustments were made to modify brightness. The photos were inverted on either the horizontal or vertical plain. Shifting or panning was also employed to add depth and texture by adjusting the pixel positions, along with embossing. These techniques collectively contributed to higher classification accuracy and improved image quality.

**Figure 6:**
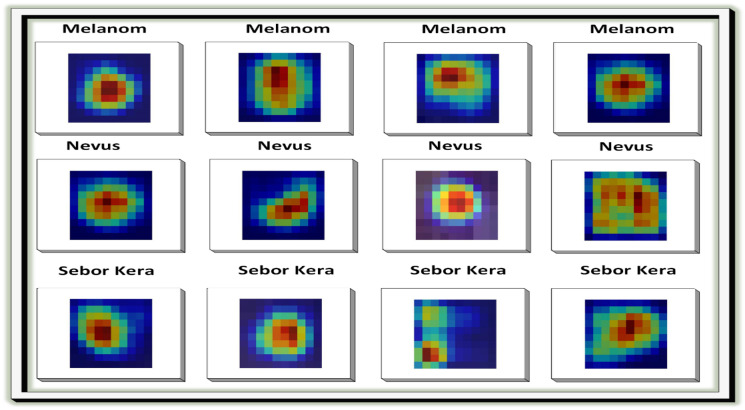
Visual result of image preprocessing using Grad-CAM technique (red represent cancer cells).

**Figure 7:**
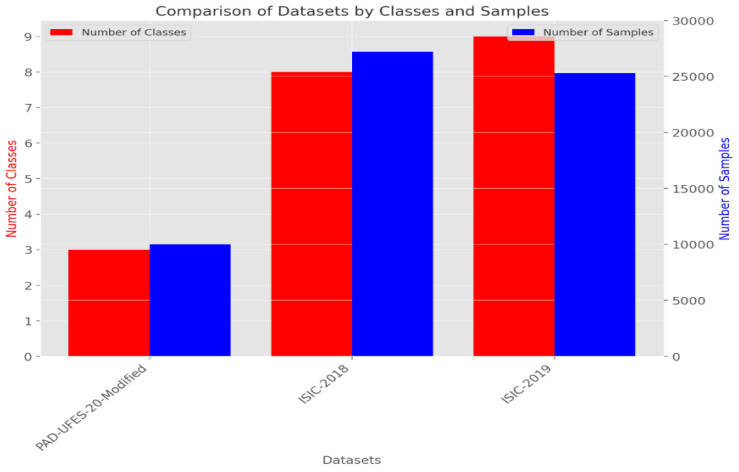
Comparison between three datasets (PAD-UFES-20-Modified, ISIC-2018, ISIC-2019).

**Figure 8:**
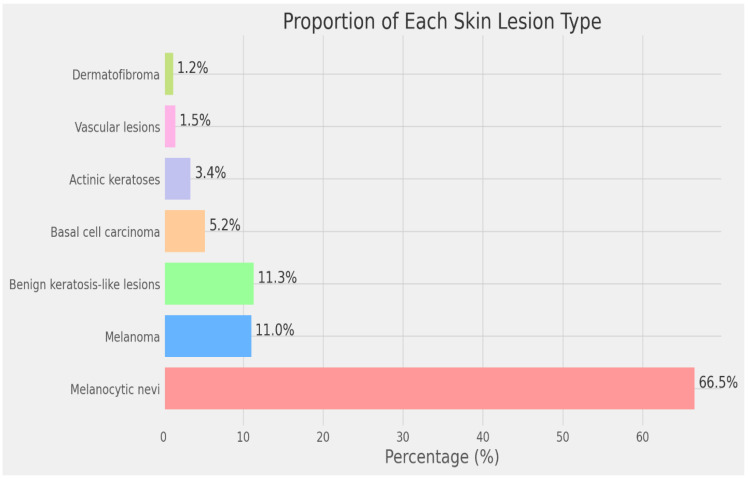
Proportion of each skin lesion type (dermatofibroma, vascular lesions, actinic keratoses, basal cell carcinoma, benign keratosis-like lesions, melanoma, melanocytic nevi).

In the next step, the images are processed using Grad-CAM (Gradient-weighted Class Activation Mapping). Grad-CAM enhances the interpretability of deep neural networks, particularly in computer vision, by generating clear heatmaps that highlight important regions for classification within an image. It has a straightforward implementation and is effective in object localization. Among various techniques for AI explainability, Grad-CAM distinguishes itself with its simplicity, focus on localization, and ability to provide clear visualizations. It has gained widespread acceptance in the deep learning community. Figure 6 illustrates the implementation of Grad-CAM. Figure 7 and Figure 8 show the dataset composition and the percentage of each form of skin lesion separately.

### 3.2. Data Augmentation

In this work, a number of datasets are combined into a new dataset that is used for training and testing the model. This new combined dataset is an imbalanced dataset that may result in a biased model. To solve this issue, Generative Adversarial Networks (GANs) are used to generate new, synthetic instances of the minority class, thus helping to balance the dataset. In the next section, the mathematics behind GANs is discussed briefly.

The basic idea of Generative Adversarial Networks (GANs) is elegantly captured by a min–max game between two distinct entities: the generator (G) and the discriminator (D). This adversarial game is mathematically formulated as $\min_{G}\max_{D}V\left(D,G\right)$, where V(D,G) represents the value function denoting the payoff of the discriminator. Specifically, this value function is composed of two expectations: $E_{x∼p_{\mathrm{data}}\left(x\right)}\left[\log D\left(x\right)\right]$, which expects the discriminator to assign high probabilities to real data, and $E_{z∼p_{z}\left(z\right)}\left[\log\left(1-D\left(G\left(z\right)\right)\right)\right]$, which expects the discriminator to assign low probabilities to fake data generated by the generator, where x represents real data samples drawn from the true data distribution p_data and z denotes noise samples drawn from a predefined noise distribution p_z. The generator, G, seeks to map these noise samples to the data space in a manner that the discriminator, D, finds indistinguishable from real data. Training a GAN involves iteratively updating the discriminator and generator in a competitive manner.

Initially, both models are defined with specific architectures suitable for the data and task at hand. Training proceeds in epochs, each comprising several batches of data. For each batch, the generator first produces fake data from random noise inputs. The discriminator then assesses both real data and fake data, updating its parameters to better differentiate between the two.

Next, the generator parameters are updated according to the feedback from discriminator, and then its ability to generate convincing samples is enhanced.

The role of the discriminator is to differentiate between real and fake data with high accuracy. On the other hand, the generator aims to make the discriminator make as many mistakes as possible. The training cycle is repeated for a specified number of epochs or until the generator produces an imputation that is of sufficiently high quality. The effectiveness of the training can be evaluated temporally by checking the quality of the generated samples in each interval. In the last instance, the measure of the success of a GAN is its generator’s capability to produce data that are conceivably real. The training objective of a GAN can be expressed as a min–max game between *D* and *G,* formulated by the value function:

(1) $$E_{x∼p_{\mathrm{data}}\left(x\right)}\left[\log D\left(x\right)\right]$$

(2) $$E_{z∼p_{z}\left(z\right)}\left[\log\left(1-D\left(G\left(z\right)\right)\right)\right]$$

(3) $$V\left(D,G\right)=E_{x∼p_{\mathrm{data}}\left(x\right)}\left[\log D\left(x\right)\right]+E_{z∼p_{z}\left(z\right)}\left[\log\left(1-D\left(G\left(z\right)\right)\right)\right]$$

where x is a real instance from the data distribution data p. z is a noise sample from distribution p_z. D(x) is the discriminator’s estimate of the probability that real data instance x is real. G(z) represents the data generated by the generator from noise z. D(G(z)) is the discriminator’s estimate of the probability that a fake instance is real. The overall process of data augmentation is shown in Table 5. The training process for a Generative Adversarial Network (GAN) is presented in a structured, tabular format. This algorithm encapsulates the iterative training loop where the generator (G) and discriminator (D) models are updated in turn.

Balancing G and D: It is crucial to maintain a balance between G and D’s learning progress. If D becomes too effective too quickly, G may fail to learn properly. Convergence: GAN training may not converge in the traditional sense. Instead, the goal is to reach a point where G generates high-quality data. Hyperparameters: Careful selection of learning rates, batch size, and architecture is essential for successful GAN training. Stability: GAN training can be unstable. Techniques like using different learning rates for G and D, gradient clipping, or employing specialized architectures and normalization techniques can help. This tabular representation provides a step-by-step overview of the GAN training process, emphasizing the adversarial training dynamics between the generator and discriminator.

### 3.3. Model Architecture and Development

The main contribution of this work is the autoencoder in the heart of our methodology. The autoencoder architecture is used to extract important features to help the deep learning model achieve high accuracy.

An encoder and a decoder are the two primary parts of an autoencoder. The encoder maps the input images to a low-dimensional latent space, and the decoder attempts to use the latent representation to create the original. The encoder E can be defined as the function(x) = z, which maps the input image x to the encoded representation z in the latent space. A mathematical representation for activation functions in a neural network modeled as a series of convolutional operations can be written as:

(4) $$Z=f(W_{e}\cdot x+b_{e})$$

where $W_{e}$ and $b_{e}$ are the weights and biases of the encoder layers and f denotes an activation function, typically ReLU f(x)=max(0,x)) for intermediate layers. The decoder *D* aims to map the latent representation *z* back to the original space, defined as:

(5) $$D\left(z\right)=\hat{e}$$

where $\hat{e}$ is the reconstructed image. Similarly, this process involves a series of convolutional and up-sampling layers, expressed as:

(6) $$X=g(W_{d}\cdot z+b_{d})$$

The decoders $W_{d}$ and $b_{d}$ represent the decoder’s weights and biases, with *g* being an activation function, which is often sigmoid for the output layer to normalize the output between 0,1,2,3,4,5,6,7,8,9. Training an autoencoder minimizes the loss function that denotes the difference between the original image *x* and the reconstructed image $\hat{e}$. A common choice is the binary cross-entropy loss, given by:

(7) $$L\left(x,\hat{e}\right)=-\frac{1}{N}\sum_{i=1}^{N}\left[x_{i}\log\left({\hat{e}}_{i}\right)+\left(1-x_{i}\right)\log\left(1-{\hat{e}}_{i}\right)\right]$$

The encoder section of the autoencoder comprises convolutional layers equipped with 3 × 3 kernels and ReLU activation functions. These layers show an accurate raster and more complex shapes with the use of a variety of applied filters. Convolutional layers are coupled with max pooling, with a 2 × 2 window size and stride of 2 for each of them. This operation performs a halving of the spatial features of the feature map by combining the extracted information, which results in the model being introduced with the translational invariance.

The reduction in dimensionality results in reducing the computational demands on the end layers and helps with the extraction of the most significant features into a compact latent representation. The decoder architecture is also as complex as the encoder, having convolutional layers that are followed by up-sample operations. The convolutional layers in the decoder essentially form a reverse encoding procedure, trying to undo the distortions introduced during the encoding phase and recover the original image from its encoded state. By up-sampling, however, the layers, which perform the operation that is a reverse process to max pooling, increase the spatial dimension of the feature matrices, paying great attention to recovering the image of its high quality and detail.

This decoder is designed to enhance the image resolution by a factor of 4×. It is this part of the framework that best illustrates the high level of adaptability of autoencoders, allowing them to perform numerous different operations, from standard encoding and decoding tasks to image quality enhancement. Besides the super-resolution capabilities, the classification head is arranged within the design of the model, and it is used to predict labels. Such a module consists of a flattening layer that turns a 2D feature map vector into a row vector, in which the objects become dense. Densely setting the ReLU activation layer learns the non-linear combinations of the features, which are finally relied upon by the softmax output layer, and the probability is distributed over the classes.

**Figure 9:**
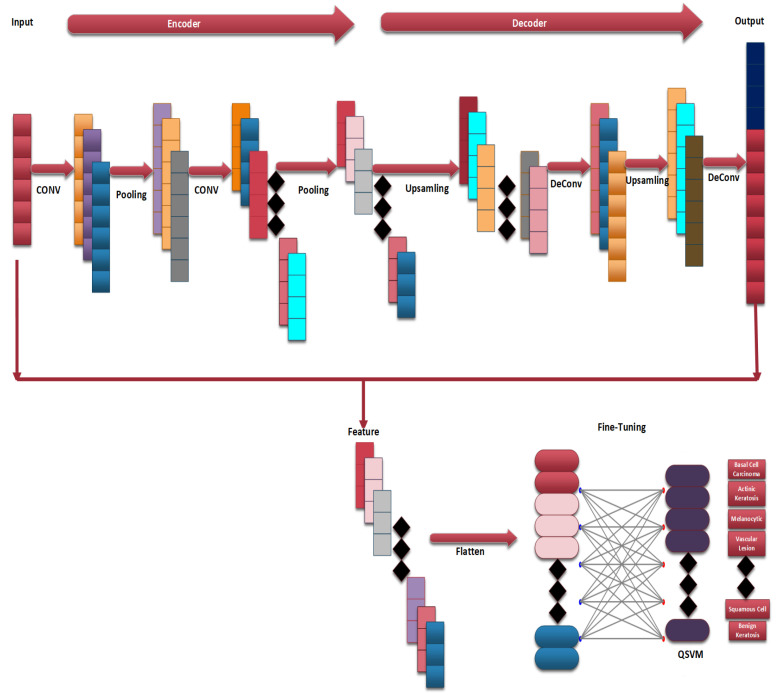
Architecture of CAD-Skin model.

**Figure 10:**
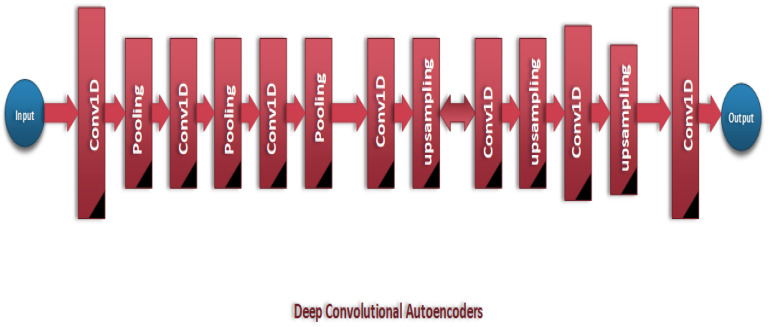
CAD-Skin model.

This method enables the model to execute multi-class classification since the encoder collects deep information that the decoder uses to carry out its assigned task, demonstrating the model’s flexibility. The images provided in Figure 9 and Figure 10 demonstrate the layout of the structure.

The average absolute distance between the original images and their reconstructed copies serves as the means for assessing the normal error in reconstruction. Anomalies are flagged if the images deviate too differently from the standard and have reconstruction errors on the higher side. This approach responds to the autoencoder’s inbuilt sensitivity to the peculiarity of data patterns; hence, any deviations that may represent important anomalies in the database are identified. Table 6 shows the training algorithm for the Autoencoder.

The proposed approach fully investigates the image processing capabilities of autoencoders. This research not only presents a case for the use of autoencoders in basic image analysis but also opens avenues for conducting future research that could challenge the limits of what can be reached in image processing using deep learning.

**Autoencoder algorithm**

The algorithm expressions and equations precisely states the key things that are involved in autoencoder training and used for tasks, which include denoising image reconstruction, feature extraction, super-resolution, as well as anomaly detection. The algorithm simulates a systematic workflow starting from the preparation of a dataset, setting the model parameters, training the model, evaluating its performance, and employing it to increase the depth of reconstructed pictures. Table 7 explicitly describes the hyperparameter settings for the proposed model, incorporating parameters such as learning rate, number of epochs, regularization, etc. This description helps us to become acquainted with the configuration of the model, giving a simple and complete picture.

### 3.4. Quantum Support Vector Machine Classifier (QSVM)

The Quantum Support Vector Machine (QSVM) is an effective classifier that is used in the last stage (classification stage) of our proposed model. The proposed QSVM classifier is based on quantum computing theories is shown in Table 8. The L2 regularization settings are used to improve the labeled version of the QSVM classifier. Subsequently, the QSVM model incorporates features from a feature map, such as Second-Order Expansion, which converts the input data into the quantum expanded feature space. When the samples are tested, the model classifies them using the quantum algorithm’s evaluation function. This is intended to facilitate a quick and accurate categorization procedure.

The classifier uses a Quantum Support Vector Machine (QSVM) to classify areas as malignant or normal. After labeling the QSVM classifier, an L2 regularization parameter is introduced for optimization. Typically, this process is described by the following equation:

(8) $$\mathrm{Optimization:}\;\arg\min_{w}\left(\frac{1}{2}{∥w∥}^{2}+C\sum_{i=1}^{l}\xi_{i}\right)$$

All of the properties $x=(z_{1},z_{2},\cdots ,z_{n})$ obtained from a feature map are used by the instantiated QSVM. The QSVM model is derived from quantum circuits, which are represented by the equation:

(9) $$\mathrm{Quantum}\;\mathrm{Circuit:}\;\exp\left(i\sum_{i=1}^{N}\theta_{i}\sigma_{i}\right)$$

where the Pauli matrices are indicated by $\sigma_{i}$ and the angles for the quantum gates are represented by $\theta_{i}$.

When testing samples are then provided, the QSVM model uses a quantum circuit to assess them and produce predictions for classification. Usually, this evaluation function is represented as:

(10) $$\mathrm{Evaluation}\;\mathrm{Function:}\;A_{\mathrm{test}}=\left(B_{\mathrm{eig}},C_{\mathrm{iv}}\right)+d$$

**Figure 11:**
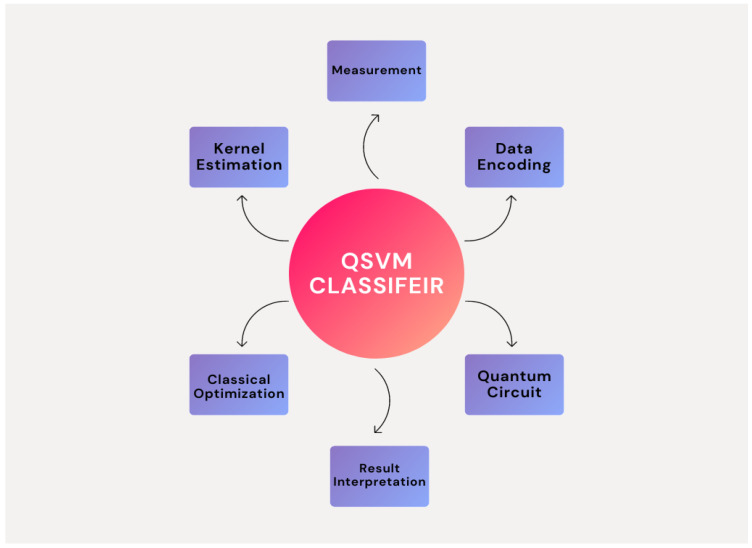
QSVM Classifier basic components.

In this case, the predicted label is represented by $A_{\mathrm{test}}$, the quantum circuit’s eigenvectors are denoted by $B_{\mathrm{eig}}$, the quantum circuit’s invariance is represented by $C_{\mathrm{iv}}$, and *d* is a constant term. These formulas show how the QSVM algorithm works well for a variety of classification tasks, making it easier to accurately classify normal and abnormal sections. Figure 11 shows the basic components of the QSVM classifier.

## 4. Results

The CAD-Skin model was trained using a dataset of 27,357 high-resolution images that included various types of skin conditions such as actinic keratosis, basal cell carcinoma, and melanoma, among others. These images were collected from several reputable online sources and were all normalized to the same pixel dimensions to help with the feature extraction and classification processes. In the first step of this work, the autoencoder architecture-based CAD-Skin model was trained for 50 epochs. The model achieved optimal performance in the 40th epoch. The F1-score at this epoch was 0.99. In the next step, statistical assessment to evaluate true reliability, specificity, and sensitivity of the model was performed. The output was compared to those of other similar systems. The experiments were running on a dual-CPU setup by Acer-i9 that incorporates 32 GB of RAM with 16 GB NVIDIA GPU via Windows 10 Professional 64-bit as an operating system.

### 4.1. Experiment 1

In this experiment, the performance of the recommended model CAD-Skin is tested using the “ISIC-2018” [56] dataset, which the authors acquired from an online internet source. First, the loss function of the model is evaluated, and the model performance on the training and validation sets is reported. In this experiment, the ISIC-2018 dataset, which includes 5190 samples of different skin diseases (actinic keratosis, basal cell carcinoma, and melanoma, among others), with clinical pictures and patient medical information is used.

**Figure 12:**
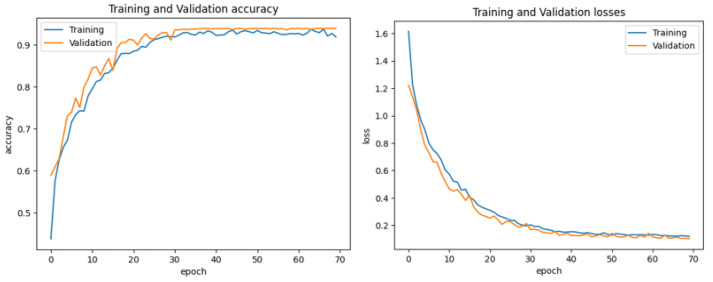
During the Skin-D model’s training, validation accuracy and loss.

**Figure 13:**
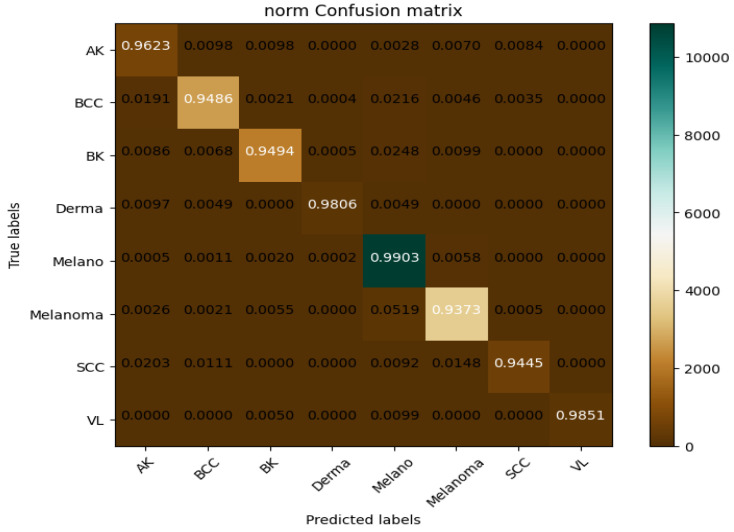
This displays the confusion matrix for ISIC-2018.

This dataset provides a real-world representation of the problem by being enhanced with a range of medical images and related medical data. The dataset was used to train the SkinNet-INIO model [53]; hence, it can be mentioned here for comparison. In the first step of this experiment, the images undergo standardization to 224 × 224 pixels. Then, the image datasets are partitioned into training, validation, and test sets to train the model to be generalized on different scenarios. Figure 12 and Figure 13 represent the training and validation accuracy of the CAD-Skin model when it was trained on this dataset. The results clearly demonstrate the effectiveness of our model in both the training and validation scenarios.

The proposed model achieved an impressive 98% accuracy rate for the training and validation datasets.

### 4.2. Experiment 2

In this experiment, the effectiveness of the CAD-Skin method using another dataset, ISIC-2019 [56], is tested. The dataset comprises nine classes (actinic keratosis, basal cell carcinoma, and melanoma, among others). The dataset was downloaded from the famous Kaggle website.

**Figure 14:**
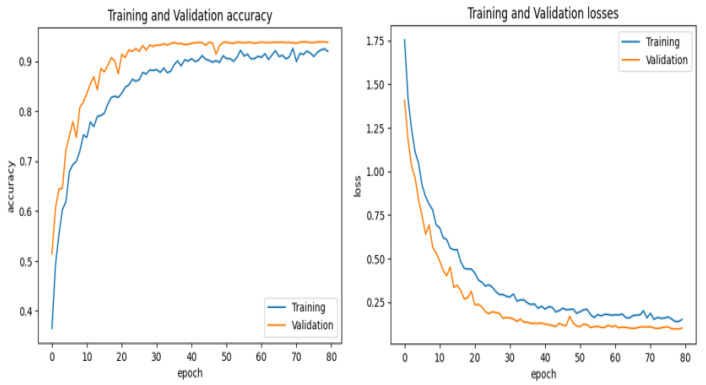
The validation accuracy and loss during the training of the CAD-Skin model.

**Figure 15:**
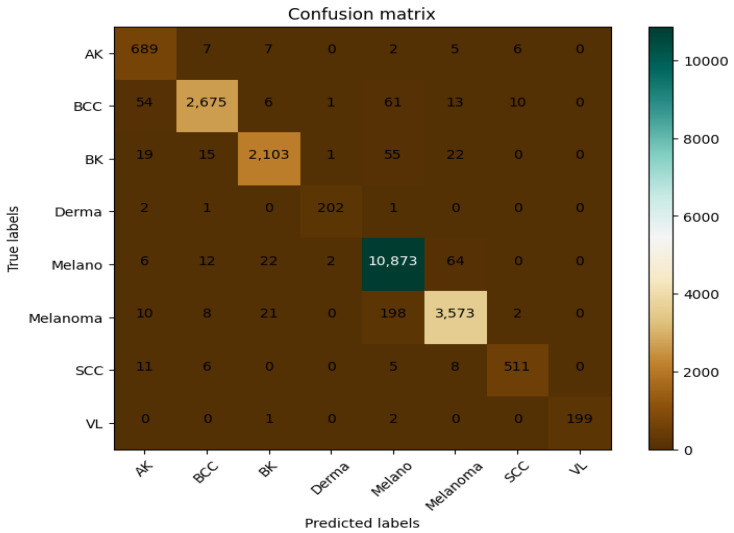
The CAD-Skin model’s confusion matrix was produced by this dataset.

The first step in this experiment is evaluating the performance of the model on the training and validation sets from the chosen dataset. Figure 14 and Figure 15 show the training and validation accuracy of the proposed model and the confusion matrix. The results prove the remarkable effectiveness of our model across both training and validation scenarios. The proposed model achieved an impressive 99% accuracy rate for the training and validation datasets.

### 4.3. Experiment 3

**Figure 16:**
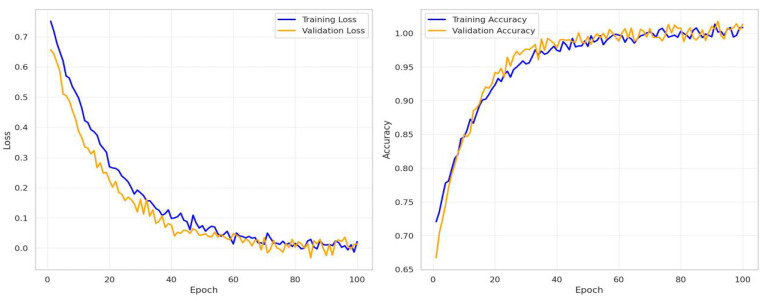
Validation accuracy and loss during the training of the CAD-Skin model.

In this experiment, the proposed CAD-Skin technique is tested using the PAD-UFES-20-Modified dataset acquired from [57]. The dataset includes 1314 samples of different skin diseases (nevus, melanoma, and seborrheic keratosis, clinical pictures) and other patient medical information. In the first step, images are standardized to 224 × 224 pixels. Then, to train and validate the model, the dataset is split into training and test sets. In the testing stage of the model, the loss function of the model is evaluated and reported for both the training and validation sets using the proposed dataset. Figure 16 shows the training and validation accuracies of the suggested novel model. The results clearly demonstrate the effectiveness of our model in both training and validation scenarios. In this experiment, the model achieved impressive 99% accuracy on the training and validation datasets.

## 5. State-of-the-Art Comparison

Table 9, Table 10 and Table 11 present the results of the comparison between the proposed CAD-Skin model with the SkinNet-INIO, MSRNet, and SkinLesNet models referenced in research articles [53,54,57].

In these previous models from the literature, the authors were able to benefit from the power of pretrained deep learning models. The authors used different techniques to increase the accuracy of these models like feature fusion techniques and the softmax classifier. The authors reported impressive accuracies of up to 96%.

**Figure 17:**
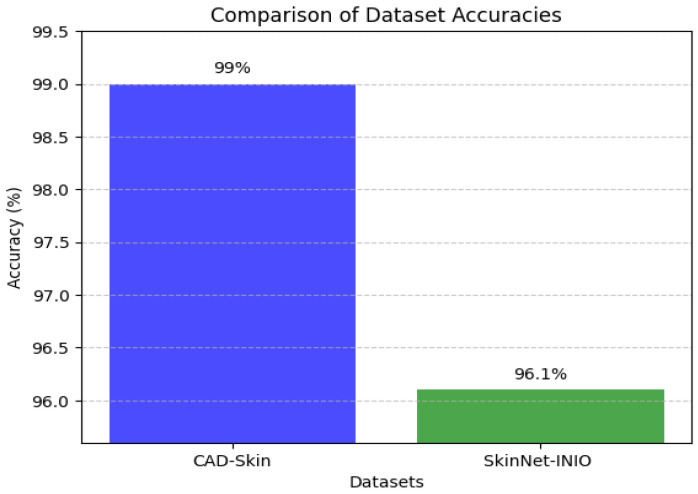
Comparison between CAD-Skin and SkinNet-INIO.

**Figure 18:**
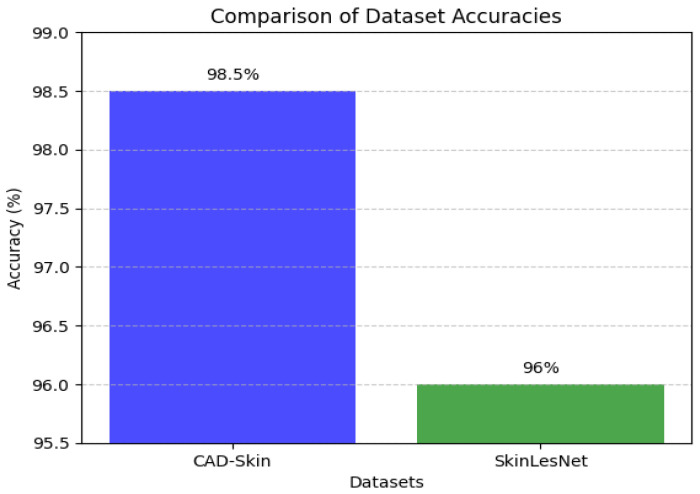
Comparison between CAD-Skin and SkinLesNet.

**Figure 19:**
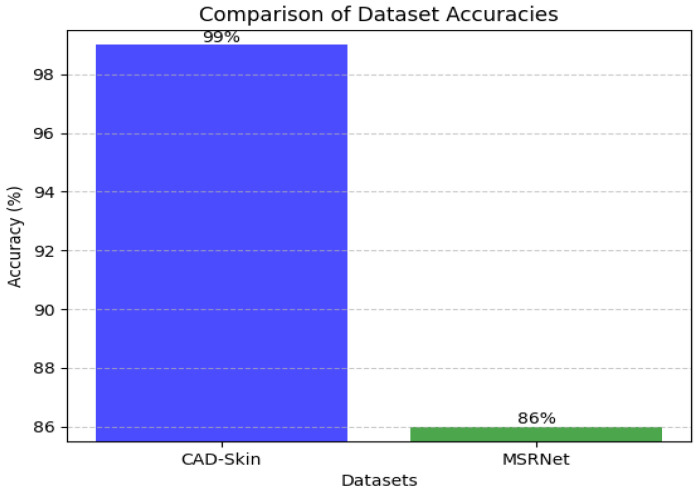
Comparison between CAD-Skin and MSRNet.

In this work, we introduce CAD-Skin, an innovative approach built using an autoencoder architecture. The proposed architecture uses the autoencoder to extract essential features from the images. The model also harnesses the power of transfer learning, allowing it to be generalized on a wide range of skin diseases. Another contribution of this work is the incorporation of a Quantum Support Vector Machine (QSVM) classifier. This innovative classifier plays a crucial role in elevating the model’s classification accuracy. As a result of these enhancements and strategic design choices, CAD-Skin achieves an exceptional classification accuracy of up to 99%. This achievement not only demonstrates the efficacy of CAD-Skin in diagnosing skin conditions but also highlights the potential for cutting-edge technologies like QSVM to revolutionize the field of medical diagnostics. Figure 16, Figure 17, Figure 18 and Figure 19 show the comparison between different models.

## 6. Discussion

The main finding of this study is that the proposed CAD-Skin framework, integrating a hybrid CNN–autoencoder architecture with a Quantum Support Vector Machine (QSVM) classifier, achieves state-of-the-art accuracy of 99% in skin lesion classification, outperforming existing deep learning-based methods. The model demonstrated superior performance across multiple benchmark datasets, including ISIC-2018, ISIC-2019, and PAD-UFES-20-Modified, effectively distinguishing between different skin disease categories such as melanoma, basal cell carcinoma, and actinic keratosis. These results highlight the effectiveness of deep feature extraction using autoencoders, quantum-enhanced classification, and advanced image preprocessing techniques, such as multi-scale retinex, gamma correction, and contrast-limited adaptive histogram equalization (CLAHE). The study also confirms that QSVM provides a more robust decision boundary, improving classification accuracy while mitigating overfitting. These findings demonstrate that CAD-Skin is a reliable AI-driven diagnostic tool, with the potential to significantly enhance early detection and classification of skin cancer in clinical settings.

Today, computer technology is being used to provide the solutions for disease identification and classification based on symptoms and other related things. These systems allow patients to diagnose diseases at early stages and increase chances of recovery. Skin diseases are a very common type of human disease that can be detected by such systems at early stages. A number of researchers are working on developing sophisticated systems using deep learning models. However, there is still a lot of effort that needs to be put forth to achieve accuracy and reliability in the diagnosis of skin diseases. Skin cancer is usually visible in upper layers of the skin, and it is also known that if diagnosed at early stages it can be fully cured.

Artificial intelligence plays a critical role in skin cancer classification and has the potential to improve healthcare services by reducing the time required for examinations. This can be crucial in situations of emergency where time is of the essence or in places where there is a scarcity of medical personnel.

Artificial intelligence algorithms can be used in the implementation of medical systems to diagnose such diseases. Also, the use of deep learning models and image processing is a very useful tool for the classification of diseases and illnesses. But to make sure they satisfy patients’ needs and work well in clinical settings, they should be utilized responsibly and cautiously in conjunction with dermatological doctors and other medical specialists [59]. These kinds of systems can reduce human error and automate the process of identifying the type of skin disease to save people’s lives.

However, the broad spectrum of diagnoses in skincare still presents a challenge for training ML models.

In recent years, the number of researchers and the development of new technologies has increased rapidly to provide solutions to identify skin conditions and face these challenges. The proposed methodologies are moving from machine learning methods to deep learning techniques. These modern deep learning methods are achieving state-of-the-art performances in various fields of study. In this work, a novel system (CAD-Skin) for skin disease classification is proposed. The CAD-Skin system achieves high accuracy in diagnosing and classifying a wide spectrum of skin diseases through sophisticated deep learning models, including convolutional neural networks (CNNs) and autoencoders. In this work, innovative data preprocessing techniques, such as perceptual algorithms based on multi-scale retinex and contrast adaptive histogram transformations, are also proposed to enhance images and increase model accuracy. The proposed model achieved 99% accuracy in classifying skin lesions, highlighting its potential future impact on clinical practice. During the investigation, the CAD-Skin system underwent extensive testing on diverse datasets, including publicly available datasets and proprietary image bases, covering the entire spectrum of skin conditions from benign lesions to malignant melanomas. The proposed model was trained and tested using the PAD-UFES-20, ISIC-2018, and ISIC-2019 datasets, widening the borders to various skin conditions to experiment with and evaluate the CAD-Skin model evaluation in both the training and the validation phase.

The major contribution of this work is proposing a new autoencoder architecture that has specifically been designed to overcome important challenges such as speed, overfitting, and accurate severity assessment in skin disease classification. Another contribution of this work is the use of diverse datasets for evaluating the model. The dataset is a combination of a number of datasets. These datasets (PAD-UFES-20-Modified, ISIC-2018, and ISIC-2019) are exceptional due to their composition of diverse clinical images as well as patient data. The chosen dataset goes through standardization and augmentation processes. Another contribution of this study is the use of the QSVM (Quantum Support Vector Machine) classifier as a final classifier. Apart from having low decision margins, the QSVM classifier also benefits from a level of robustness and interpretability that was not achieved in previous skin disease classification studies. This means that the proposed study has followed cutting-edge methodology as it involves the combination of classical deep learning techniques with quantum computing principles to reach the high level of a disease severity classification. With the thoughtful merging of autoencoder structure, sophisticated dataset preparation, and adaptive preprocessing, the implementation of this quantum classifier made an impact on the quality, efficiency, and practicality of the classifications of the diseases of skin. The obtained outcomes not only support the idea of using the proposed model CAD-Skin solution but also bring the possibility of the integration of quantum computing classification into medical diagnostics.

Most of the work introduced in Table 2 shows low accuracy in some articles, around 86%, which is very low compared to the proposed model in this work. The model proposed in this work reaches very high accuracies and generalizes very well on different datasets. This is the main advantage, despite the fact it is more computationally expensive, especially with large datasets. However, this point can be challenged with more speedy GPUs to run the algorithm. The proposed model was tested using a number of experiments to compare it with state-of-the-art models working on the same application. All experiments and results prove the superior performance of the proposed model.

### Limitations

While the CAD-Skin framework demonstrates high classification accuracy (99%) and outperforms existing deep learning-based models, it has certain limitations that need to be addressed in future research. One major limitation is the dependency on publicly available datasets (ISIC-2018, ISIC-2019, and PAD-UFES-20-Modified), which may not fully represent the diversity of real-world clinical cases. The datasets primarily contain images from specific geographic regions, and underrepresentation of certain skin tones and lesion types could affect the model’s generalizability. Another limitation is the computational complexity of the Quantum Support Vector Machine (QSVM) classifier. While the QSVM offers superior classification performance, its training and inference times are higher compared to traditional classifiers, making it challenging to deploy in real-time clinical settings. Future work should explore hardware acceleration techniques or hybrid quantum-classical models to improve efficiency. Furthermore, although image preprocessing techniques such as multi-scale retinex, gamma correction, and CLAHE improve model robustness, they may introduce artifacts in certain cases, potentially affecting feature extraction. An adaptive preprocessing approach tailored to different skin types and lighting conditions could enhance the model’s reliability. Finally, while CAD-Skin performs well in multi-class classification, it currently lacks an uncertainty quantification mechanism to indicate cases where the model is uncertain about its predictions. Integrating confidence-aware AI techniques or explainability methods such as SHAP or LIME could further improve its clinical usability. Addressing these limitations will help refine the CAD-Skin system and make it more suitable for real-world deployment in dermatological diagnostics.

## 7. Conclusions

This study proposed CAD-Skin, a hybrid CNN–autoencoder framework with a Quantum Support Vector Machine (QSVM) classifier for accurate skin lesion detection and classification. The model achieved state-of-the-art accuracy of 99% on the ISIC-2018, ISIC-2019, and PAD-UFES-20-Modified datasets, outperforming existing deep learning methods. The integration of advanced image preprocessing techniques (multi-scale retinex, gamma correction, and CLAHE) enhanced feature extraction, while autoencoders improved deep feature representation. QSVM further strengthened classification by providing superior decision boundaries and robustness against overfitting. The proposed system has significant potential for clinical deployment, including applications in telemedicine, mobile health (mHealth) self-screening, and AI-assisted dermatology. Despite its success, the model’s computational complexity and dataset diversity limitations highlight the need for future improvements, such as quantum computing implementations, adaptive preprocessing techniques, and uncertainty-aware AI models. In summary, CAD-Skin presents an innovative, highly accurate, and scalable solution for automated skin cancer diagnosis, paving the way for early detection, improved clinical decision-making, and enhanced patient care.

The future scope of this research extends towards enhancing the CAD-Skin system for real-world clinical applications. One potential advancement is integrating the model into a web-based or mobile diagnostic tool, allowing dermatologists and patients to receive real-time AI-driven skin lesion analysis. Additionally, expanding the model to 3D skin lesion analysis using Optical Coherence Tomography (OCT) or multi-modal imaging can improve diagnostic precision. Incorporating Explainable AI (XAI) techniques like Grad-CAM, SHAP, and LIME will enhance model interpretability, increasing trust in AI-driven decisions. Further, the Quantum Support Vector Machine (QSVM) can be adapted for quantum hardware, leveraging platforms like IBM Q for accelerated processing and improved classification performance. To ensure clinical applicability, multi-center clinical trials should be conducted on diverse populations, addressing bias and improving generalizability. Enhancing data augmentation using GANs and self-supervised learning can further strengthen the model’s ability to classify rare skin lesions with limited training data. Additionally, integrating CAD-Skin with IoT-enabled smart dermatology systems and wearable sensors can enable continuous monitoring of skin conditions, making AI-driven dermatological care more accessible and efficient. These advancements will further refine CAD-Skin, making it a clinically deployable AI system for accurate and early skin cancer detection.

## Figures and Tables

**Table 1:** Skin disease findings as per severity level.

Severity Level	Condition	Description
0	Actinic keratosis	Precancerous skin lesion due to sun exposure; it may evolve into squamous cell carcinoma if untreated.
1	Basal cell carcinoma	The most prevalent type of skin cancer, it grows slowly and has little chance of spreading.
2	Benign keratosis	Noncancerous skin growths, including seborrheic keratoses, usually harmless and requiring no treatment.
3	Dermatofibroma	A benign, usually harmless skin growth, most often found on the legs.
4	Melanocytic nevus	Often referred to as a mole, these are benign growths that require monitoring for signs of melanoma.
5	Melanoma	This type of skin cancer is the most dangerous because it can spread quickly throughout the body.
6	Squamous cell carcinoma	A type of skin cancer that can grow and spread more quickly than basal cell carcinoma, potentially dangerous.

**Table 2:** Overview of classification techniques based on deep learning.

Reference	Methods	Datasets	Accuracy	Limitations
[32]	Deep neural network	Private	97.1%	Although highly accurate, deep neural networks require large amounts of labeled data and substantial computational resources. Their use on a private dataset also raises questions about the generalizability of the model to other datasets.
[34]	AlexNet, VGG16	Skin cancer	96%	These models, while foundational in the field of deep learning, are relatively simple and deep architectures that can lead to overfitting if not properly regularized. They also require significant computational power and memory.
[35]	ResNet, DenseNet	ISIC (2016, 2017, 2018)	88%	Gradually vanishing is well managed by these networks with the advances in deeper network architectures, but these networks are harder to train and waste resources. The reason why the diverse images do not appear in the dataset of the skin images through the lesions is the 88% precision that the network can make.
[36]	SE Net	ISIC 2019	91%	SENet consists of the feature re-shaping block using quantum-linear-time squeezing, which can incur a higher number of computations and a more complicated model. The model evaluation depends, to some extent, on the data used and does not apply in non-controlled settings.
[37]	InceptionV3 and MobileNet	Skin cancer	86%	Such models may be designed in the most efficient and effective way, but they may sometimes be too simple. This “underfitting” situation can trap some models, often in complex images, which include medical imaging. Secondly, the results of the candidates’ sensitivity and specificity possess an accuracy of only 86%.
[38]	DenseNet and ReseNet	ISIC 2017	87%	Despite their drawbacks, older architectures can be more suitable for deep networks due to their specialized design capabilities for specific datasets. However, these models have high computational requirements, and fine-tuning them can be challenging.
[39]	Linear Support Vector Machine (SVM) and Feedforward Neural Network (FNN)	Dermo fit	90%	SVM and FNN might not capture complex patterns as effectively as more sophisticated networks. The linear SVM, in particular, may struggle with non-linear decision boundaries typical in image data.
[40]	ResNet CNN	PH2	90%	ResNet generally does a good job, yet it demands careful settings of the parameters not to overfit. It performs this well for more extensive or complex datasets, but for smaller and less diverse sets like PH2 it may not adapt well.
[42]	MobileNetV2	HAM1000	92.4%	One feature that distinguishes MobileNetV2 from others is that it is lightweight, i.e., it was built with a focus on device performance on mobile phones. It yields excellent results when applied to the HAM1000 dataset, but it might not manage to tackle the other images that belong to more complex and diverse classes.
[43]	CNN, Soft-Max	DermNet	98.6%	These approaches may be limited by their lack of depth and complexity needed to handle a variety of dermatological conditions. Nonetheless, they demonstrate high accuracy on the DermNet dataset, which is a comprehensive dermatology database for public health purposes.
[44]	Transfer Learning, deep learning pretrained models	Private	94.1%	This approach results in supplementary training and, thus, performance superiority on small datasets. Then again, it has the potential to pass the bias from initial data, which comes to the consideration of the effectiveness of the approach on private datasets.
[49]	VGG16, AlexNet	-	90%, 95%	Older architectural models are generally less efficient than newer ones, which can result in longer training times. These outdated architectures may also consume more memory, potentially leading to increased system resource usage.
[53]	Serial Correlation-based Approach, deep learning models	ISIC2018, ISIC2019	96.1%	Although this method is innovative, its reliance on serial correlation may fail to capture non-linear relationships in data. This limitation could restrict its applicability in various scenarios where complex data interactions are crucial.
[54]	MSRNet, DarkNet-53, and DensNet-201	ISIC2018, ISIC2019	85%, 98.80%	Models like DarkNet-53 are infrequently used in medical vision, which complicates optimization efforts. This lack of common usage can also make generalization across different medical imagery datasets challenging.
[55]	YOLOv7	ISIC 2017	86.3%	The ISIC collection, comprising 2750 photos, may not fully capture the diversity of skin lesions found worldwide. This limitation could hinder the dataset’s representativeness and impact the generalizability of research findings.

**Table 3:** Three different datasets’ representation.

Reference	Name	Classes	Images	Train/Test Ratio	Image Dimensions
[57]	PAD-UFES-20-Modified	03	10.0 k	7500/2500	Vary in resolution, size, and lighting conditions
[56]	ISIC-2018	08	27.2 k	20,400/6800	1024 × 1024 pixels
[58]	ISIC-2019	09	25.3 k	18,975/6325	1024 × 1024 pixels

**Table 4:** Detailing the mathematical details and implementation methods for each of the preprocessing techniques mentioned: multi-scale retinex, gamma correction, histogram equalization, unsharp masking, and contrast-limited adaptive histogram equalization (CLAHE).

Preprocessing Technique	Mathematical Details	Implementation Method
Multi-scale retinex	*R (x,y)* = $\log\left(I(x,y)\right)-\log\left(G(x,y)\right)$	Decompose the image into multiple scales using a Gaussian pyramid. Apply retinex on each scale image.
Gamma correction	$O=L^{\gamma }$	Apply a power-law function to each pixel intensity value, where *I* is the input intensity and γ is the gamma value.
Histogram equalization	$O=T\left(I\right)=\frac{L-1}{MN}\sum_{i=0}^{L-1}n_{i}$	Calculate the histogram of the input image. Compute the cumulative distribution function. Map each pixel intensity to its new value using the CDF.
Unsharp masking	O=I+K.(I−G)	Apply a Gaussian filter *G* to the input image. Subtract the smoothed image from the original. Multiply the result by a factor k and add it back to the original image.
Contrast-limited adaptive histogram equalization (CLAHE)	$O\left(x,y\right)=\mathrm{clip}\left(\frac{I\left(x,y\right)-V_{\min}}{V_{\max}-V_{\min}}\times L,0,L-1\right)$	Divide the image into tiles. Apply histogram equalization to each tile. Interpolate the histograms of neighboring tiles. Clip the resulting pixel intensities.

*I* represents the input image. *O* represents the output image. *G* denotes the Gaussian filter. *L* is the number of intensity levels (typically 256 for 8-bit images). *M* and *V* represent the dimensions of the image. *ni* is the number of pixels with intensity in the input image. *Vmin* and *Vmax* are the minimum and maximum values in the neighborhood of a pixel, respectively. *k* is a scaling factor for the unsharp masking operation. *x* and *y* represent pixel coordinates.

**Table 5:** Data augmentation algorithm steps.

Step	Action	Description
1	Initialization	Initialize the generator (G) and discriminator (D) models with the chosen architectures. Define the noise distribution $p_{z}\left(z\right)$. Select hyperparameters: learning rates, batch size, and the number of epochs.
2	For each epoch	Repeat the following steps for a specified number of epochs or until *G*’s output is satisfactory.
3	Generate data	Sample a minibatch of *m* noise samples $\left\{z^{1},\ldots ,z^{m}\right\}$ from the noise distribution $p_{z}\left(z\right)$. Use *G* to generate a minibatch of fake data $\left\{x_{\mathrm{data}}^{1},\ldots ,x_{\mathrm{data}}^{m}\right\}$ from these noise samples.
4	Train discriminator (*D*)	Compute *D*’s loss on both real data logD(x) and fake data log(1−D(G(z))). Update *D* by ascending its stochastic gradient to maximize its ability to distinguish real from fake data.
5	Train generator (*G*)	Generate a new set of fake data. Compute *G*’s loss using log(1−D(G(z))), focusing on misleading *D*. Update *G* by descending its stochastic gradient to minimize this loss, improving its ability to generate realistic data.
6	Monitoring	Optionally, generate images from fixed noise vectors at regular intervals to visually monitor *G*’s progress.
7	Evaluation	Upon completion, evaluate *G*’s performance qualitatively by examining the images it generates and/or quantitatively using metrics like Inception Score (IS) or Fréchet Inception Distance (FID), if applicable.

**Table 6:** Algorithm specifying the key steps of autoencoder training.

Steps	Descriptions	Details
1	Data Preparation	Load images from a directory. Resize images to 256 × 256 pixels. Normalize pixel values to [0,1,2,3,4,5,6,7,8]. Split into training and validation sets.
2	Model Initialization	Initialize encoder with convolutional and max pooling layers. Initialize decoder with convolutional and up-sampling layers. Define the loss function (e.g., categorical cross-entropy).
3	Training Loop	For each epoch, complete the following: **Forward Pass**: Encrypt and decrypt images. **Compute Loss**: Calculate the loss between the original and reconstructed images. **Backward Pass**: Use an optimizer to update the model weights.
4	Evaluation	Use the loss function for evaluating the model on the validation set. Optionally, calculate additional metrics (e.g., accuracy) if relevant.
5	Anomaly Detection (Optional)	For each image in a separate dataset, calculate the reconstruction error. Flag images with errors above a certain threshold as anomalies.

**Table 7:** Hyperparameter settings for the model.

Hyperparameter	Settings
Learning rate	0.001
Number of epochs	50
Batch size	32
Optimizer	Adam
Loss function	Categorical cross-entropy
Regularization	L2 regularization (0.01)
Dropout rate	0.5
Activation function	ReLU
Architecture	Autoencoder
Preprocessing	Multi-scale retinex, gamma correction, histogram equalization, unsharp masking, CLAHE
Augmentation	Geometric transformations
Classifier	Quantum Support Vector Machine
Quantum Kernel Type	Polynomial
Quantum Kernel Degree	3
Quantum Kernel Scale	1.0
Quantum Kernel Offset	0.0

**Table 8:** Quantitative Support Vector Machine steps.

Steps	Explanation
**Step 1**	**Label Classifier and Regularize L2 Parameters:**
Input:	Extracted feature map $x=(z_{1},z_{2},\cdots ,z_{n})$
Output:	Optimized QSVM classifier and kernel parameters
**Step 2**	**Create QSVM Model:**
Input:	Attributes $x=(z_{1},z_{2},\cdots ,z_{n})$ obtained from previous Algorithms
Output:	Trained QSVM model. By quantum circuits, a hyperplane would be generated.
**Step 3**	**Assign Class Label for Testing Samples:**
Input:	Testing samples $z_{\mathrm{test}}$
Output:	Normal and aberrant portions are categorized using the evaluation function of the quantum circuit: $A_{\mathrm{test}}=\left(B_{\mathrm{eig}},C_{\mathrm{iv}}\right)+d$

**Table 9:** State-of-the-art comparison of SkinNet-INIO and Skin-D using ISIC-2018.

Model	Sensitivity	Specificity	Accuracy
SkinNet-INIO [53]	96%	94%	96.1%
CAD-Skin	97%	98.5%	99%

**Table 10:** State-of-the-art comparison of CAD-Skin and SkinLesNet using the PAD-UFES-20-Modified dataset.

Model	Precision	Recall	F1-Score	Accuracy
SkinLesNet [57]	97%	92%	92%	96%
CAD-Skin	97%	97.54%	98%	98.5%

**Table 11:** State-of-the-art-comparison of CAD-Skin and MSRNet using ISIC-2018.

Model	Precision	Recall	F1-Score	Accuracy
MSRNet [54]	80%	61%	69.2%	86%
CAD-Skin	98%	99%	98.5%	99%

## Data Availability

For experimental purposes, publicly available datasets were obtained from the following sources: https://doi.org/10.3390/cancers16010108, arxiv.org/abs/1902.03368, and arXiv:1908.02288.
